# The Beat

**DOI:** 10.1289/ehp.120-a108b

**Published:** 2012-03-01

**Authors:** Erin E. Dooley

## Soil Fungi May Help in Lead Remediation

Lead contaminates soil in many urban areas. Investigators have discovered that lead can be transformed by multiple soil fungi into a stable form known as chloropyromorphite, suggesting a potential new tool for cleaning up soil lead contamination.^1^ This ability of the fungi may help them to survive in contaminated locations. The authors write that “fungal metabolites, particularly organic acids, [may play] an important role in the liberation of mobile lead species,” a discovery that “may be important in other environmental contexts outside those reported in this paper.”^1^

**Figure:**
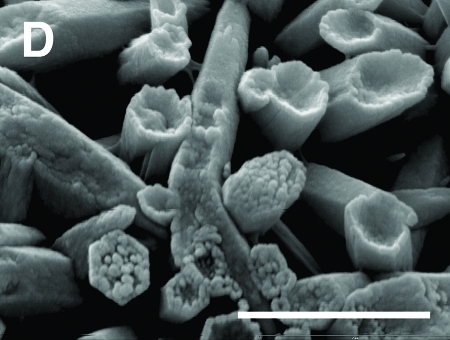
Chloropyromorphite formation on lead shot surface after incubation with the soil fungus *Metarhizium anisopliae*. Current Biology/Elsevier

## Triple A Test for Fewer Asthma Attacks

The nonprofit Asthma UK has developed a new online test to help asthma sufferers over the age of 12 predict their risk of a serious attack.^2^ The Triple A (“Avoid Asthma Attacks”) Test asks 13 questions about factors such as prior hospital admissions, allergies, and inhaler use, and explains why this information is useful in assessing the risk for an asthma attack. The Triple A website also offers guidelines for minimizing risk of asthma attacks. Asthma UK estimates that, in the United Kingdom, 75% ofhospital admissions for asthma and 90% of asthma deaths are preventable.

## Plasma Approach to Foodborne Pathogens

*Campylobacter* and *Salmonella* cause hundreds of thousands of cases of foodborne illness in the United States each year.^3^ A new proof-of-concept study shows that nonthermal plasma can be used to kill these pathogens on raw chicken without altering the texture or appearance of the meat.^4^ The study builds on earlier research that showed plasma was effective at reducing pathogens on the surfaces of fruits and vegetables. Plasma technology is not yet developed for wide use in the meat-processing industry; challenges involve devising an efficient way to fully expose all the surfaces of a piece of meat.

**Figure:**
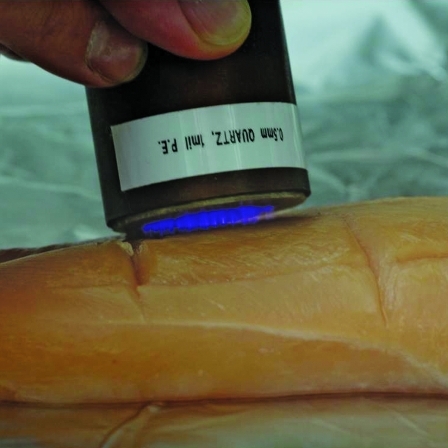
Nonthermal plasma, although similar to a gas, is a distinct state of matter containing charged particles. Brian Dirks/Drexel.edu

## Growing Counterfeit Pesticide Trade in Europe

The European Union’s law enforcement agency, Europol, estimates that as much as 25% of the pesticides used in some EU member states may originate in an illegal and counterfeit pesticide market worth billions of dollars.^5^ These cut-rate fakes may contain banned hazardous substances that make them harmful not only to human health but to crops as well.^6^ Europol and national experts have outlined a set of overarching recommendations in this area, including assessment of existing relevant legislation, coordinated cross-border investigations, adoption of a wide-ranging response to address the many potential health threats posed by counterfeit pesticides, and research on the traceability of hazardous chemicals used in these products.

**Figure:**
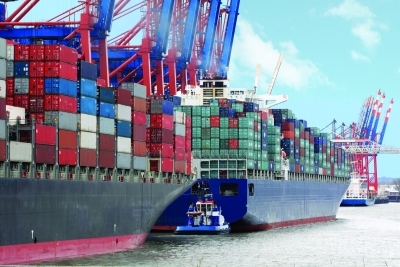
The port of Hamburg, Germany, where 28 metric tons of counterfeit pesticides were seized in 2010. Shutterstock.com

## Use of Treated Wastewater to Meet Drinking Water Demands

Amidst continued population growth, communities are searching for new ways to meet their water needs. A report by the National Research Council outlines how treated wastewater can be used to meet many of those needs, including that for drinking water.^7^ In many cases, treated wastewater is fully as safe for consumption as existing drinking water sources, or even safer. Wastewater reclamation programs are already in use in many areas, but potable applications account for only a small fraction of these programs’ activities; however, many drinking water treatment plants draw on water supplies fed by wastewater discharged by upstream communities.

## References

[1] Rhee YJ (2012). Lead transformation to pyromorphite by fungi.. Curr Biol.

[2] Triple A Test: Avoid Asthma Attacks [website]. London, UK:Asthma UK (2012). Available: http://triplea.asthma.org.uk/ [accessed 8 Feb 2012].

[3] CDC http://www.cdc.gov/foodsafety/facts.html.

[4] Dirks BP (2012). Treatment of raw poultry with nonthermal dielectric barrier discharge plasma to reduce Campylobacter jejuni and Salmonella enterica.. J Food Protect.

[5] Europol https://www.europol.europa.eu/content/press/europol-warns-growing-trade-counterfeit-pesticides-worth-billions-euros-year-1237.

[6] ECPA http://www.youtube.com/watch?feature=player_embedded&v=YJxxTkd7Xhk#!.

[7] National Research Council (2012). Water Reuse: Potential for Expanding the Nation’s Water Supply Through Reuse of Municipal Wastewater.. Washington, DC:The National Academies Press.

